# Effectiveness of an Integrated Pest Management Intervention in Controlling Cockroaches, Mice, and Allergens in New York City Public Housing

**DOI:** 10.1289/ehp.0800149

**Published:** 2009-04-15

**Authors:** Daniel Kass, Wendy McKelvey, Elizabeth Carlton, Marta Hernandez, Ginger Chew, Sean Nagle, Robin Garfinkel, Brian Clarke, Julius Tiven, Christian Espino, David Evans

**Affiliations:** 1 New York City Department of Health and Mental Hygiene, New York, New York, USA; 2 Columbia Center for Children’s Environmental Health, Columbia University Mailman School of Public Health, New York, New York, USA; 3 New York City Housing Authority, New York, New York, USA

**Keywords:** allergen, asthma, cockroach, integrated pest management, mouse, New York City, pesticide, public housing

## Abstract

**Background:**

Cockroaches and mice, which are common in urban homes, are sources of allergens capable of triggering asthma symptoms. Traditional pest control involves the use of scheduled applications of pesticides by professionals as well as pesticide use by residents. In contrast, integrated pest management (IPM) involves sanitation, building maintenance, and limited use of least toxic pesticides.

**Objectives:**

We implemented and evaluated IPM compared with traditional practice for its impact on pests, allergens, pesticide use, and resident satisfaction in a large urban public housing authority.

**Methods:**

We assigned IPM or control status to 13 buildings in five housing developments, and evaluated conditions at baseline, 3 months, and 6 months in 280 apartments in Brooklyn and Manhattan, in New York City (New York). We measured cockroach and mouse populations, collected cockroach and mouse urinary protein allergens in dust, and interviewed residents. All statistical models controlled for baseline levels of pests or allergens.

**Results:**

Compared with controls, apartments receiving IPM had significantly lower counts of cockroaches at 3 months and greater success in reducing or sustaining low counts of cockroaches at both 3 and 6 months. IPM was associated with lower cockroach allergen levels in kitchens at 3 months and in beds and kitchens at 6 months. Pesticide use was reduced in IPM relative to control apartments. Residents of IPM apartments also rated building services more positively.

**Conclusions:**

In contrast to previous IPM studies, which involved extensive cleaning, repeat visits, and often extensive resident education, we found that an easily replicable single IPM visit was more effective than the regular application of pesticides alone in managing pests and their consequences.

Cockroaches and rodents are present in the homes of many urban residents in the United States. Besides causing annoyance and stress, they are sources of allergens that can trigger asthma symptoms in sensitized individuals, and they may increase the risk of allergic sensitization (Chew et al. 2008; Gruchalla et al. 2005; Huss et al. 2001; Matsui et al. 2004; Morgan et al. 2004; Nelson 2000; Phipatanakul et al. 2000; Rosenstreich et al. 1997).

The professional control of residential pests has traditionally depended on personnel licensed by states to apply pesticides. Pesticides applied in urban homes include insecticides and rodenticides, and their chemical formulations frequently involve active and inactive ingredients that are acute toxicants, known or suspected carcinogens (California Environmental Protection Agency 2005), or developmental or reproductive toxicants [U.S. Environmental Protection Agency (EPA) 2004]. Their application may result in human exposure via inhalation, ingestion, or skin absorption from initial applications and their residual presence.

Pest control that depends on chemical-only approaches is limited by its failure to address conditions that sustain pest populations—the ability of pests to move within and between residences, the presence of food and water sources typically found in kitchens and bathrooms, and the ability of pests to find and create shelter. Integrated pest management (IPM) is an approach that primarily involves improving sanitary and structural conditions to deny pests food, water, harbor-age, and movement, and includes the judicious use of pesticides after an evaluation of need and the hazard to human occupants.

Several studies have evaluated the use of interventions in which residents were given equipment and taught how to use IPM principles to control cockroaches and allergens in their homes, either alone (Klinnert et al. 2005; Krieger et al. 2005; McConnell et al. 2005) or in combination with professional pest control using low-toxicity pesticides (Brenner et al. 2003; Condon et al. 2007; Morgan et al. 2004; Peters et al. 2007). These studies suggest that education about IPM, either alone or combined with commercial cleaning, successfully reduced either cockroach counts (Brenner et al. 2003; McConnell et al. 2005) or cockroach allergen levels (Klinnert et al. 2005; McConnell et al. 2005; Morgan et al. 2004). Another study compared IPM that included repeated visits with the use of insect growth regulator devices to treatment with spray pesticide alone in public housing, using a commercial service for both treatments (Miller and Meek 2004). The results suggested that IPM was superior to the traditional intervention in reducing cockroach populations. Two features of these studies limit their instructiveness for significant institutional replication and expansion. First, these studies have typically recruited from, and analyzed impact of IPM on, individual rental apartments without building-level interventions. Second, these studies, some of which involved goals beyond pest control, have involved repeated visits by pest control professionals, cleaning services, or educators. The present study, in contrast, involves a single visit by pest control professionals with the intervention at the building level.

The New York City Housing Authority (NYCHA) is the largest public housing owner in North America, with > 405,000 low-income residents. The successful implementation of IPM in an institution of this size was thought to offer many potential benefits: pesticide use reduction, improved pest management, and reduced pest and allergen burdens in housing populated by largely black and Hispanic families with disproportionately high prevalence of asthma (Stevenson et al. 2000). After a successful pilot program in public housing (Kass and Outwater 2003), the New York City Department of Health and Mental Hygiene (DOHMH) and the NYCHA developed an IPM intervention designed to be simple, low cost, and relatively easily scaled if successful. It involved a single visit to homes by a specially constituted team of NYCHA pest control personnel during which IPM services were conducted in kitchens and bathrooms. This intervention was compared with the NYCHA’s conventional pest control, which involved the offer of calendar-based application of spray pesticides in nonintervention apartments, for its impact on populations of cockroaches and mice and levels of cockroach and mouse allergen.

## Methods

### Study design

The NYCHA owns and manages 178,000 apartments in 343 developments within NYC, each of which has one or more residential buildings. We conducted an intervention study in 13 buildings or building groups owned within 5 developments. A convenience sample of housing developments was selected that met three criteria: location in a neighborhood with a rate of asthma hospitalization higher than the citywide average; good structural condition of buildings with no major capital renovations planned for several years; and the presence of an active residents association (RA). The selected developments were located in Bushwick, Brooklyn, and East Harlem, Manhattan.

Within housing developments, we assigned buildings to IPM intervention or control status. IPM status was assigned to whole buildings to facilitate efficient service delivery and out of a belief that pests may be more successfully controlled in a given apartment if simultaneously addressed in neighboring apartments. The building sample included seven high-rises (five general population, two senior citizen only) and six three-story attached townhouses (general population). Eight of these buildings were assigned to the IPM intervention and five to control; one of the two senior buildings was assigned intervention and one control status. Control buildings were selected so as to be as physically distant as possible from intervention buildings within the developments.

Before beginning the intervention, program representatives met with RAs to discuss the study and solicit input. RA members served as liaisons between residents and the IPM and evaluation teams, answering residents’ questions about the program and providing informal feedback to the teams. The study protocol was approved by the Institutional Review Boards of Columbia University Medical Center and DOHMH, and all participants provided written, informed consent prior to the study.

### Sampling and recruitment

We randomly selected 50% of the apartments in each studied building to participate in the study, totaling 516 apartments. Residents of selected apartments were recruited through RA meetings, staffed tables in building foyers, telephone calls, and door-to-door canvassing. In an attempt to reach working residents, telephone calls were made during the day and in the evening, and evaluation visits were scheduled Mondays through Saturdays. We enrolled and completed baseline evaluations in 323 apartments (63%). Residents in 137 apartments (27%) refused to participate, 25 (5%) were never home, 10 (< 2%) were not capable of providing informed consent, 10 (< 2%) were erroneously given the IPM intervention before the baseline visit could be conducted, and the remaining 11 (< 2%) were excluded because of vacancy status or other reasons.

### Data collection

Baseline evaluations were conducted by an evaluation team in East Harlem between August and November 2002, and in Bushwick between October 2003 and May 2004. Trained research workers fluent in English and Spanish from the Columbia Center for Children’s Environmental Health and the DOHMH conducted home visits in which they interviewed heads of households in their language of choice and collected vacuumed dust samples in the kitchen and bedroom. A translator was provided by the NYCHA for several Chinese-speaking residents. The research workers also placed cockroach- and rodent-monitoring devices, which were recovered from the home 1 week later. Follow-up evaluations for all baseline measures were conducted 3 months and 6 months after the date of the IPM visit for intervention apartments, and 3 months and 6 months after the baseline evaluation visit for control apartments.

### Intervention

All apartments in intervention buildings received IPM whether or not they were selected for participation in the study. The NYCHA recruited, hired, and trained nine public housing residents to become pest control technicians in a dedicated unit to carry out IPM in intervention buildings. The staff operated in teams of three, each overseen by an NYCHA supervisor trained in IPM. The teams scheduled appointments with residents and completed IPM in a single visit that averaged 2–3 hr (8–12 person-hours). The teams employed mechanical and steam cleaning using soap (Simple Green; Sunshine Makers Inc., Huntington Beach, CA) on kitchen cabinets, stoves, refrigerators, floors, and countertops, and on bathroom floors and fixtures; they used latex caulk to seal cracks and crevices, gaps within kitchen cabinets and between the cabinets and wall, gaps and cracks in baseboards, plumbing joints, and other potential ports of entry for pests; and they applied boric acid and cockroach baits (gels or containerized solids containing fipronil or hydramethylnon) to kill remaining cockroaches. The supervisor instructed apartment residents to store open food in sealed containers, cover garbage containers with a tight-fitting lid, and dispose of garbage frequently. Residents were provided with a covered garbage container, food storage containers, and cleaning supplies, including sponges, soap, powdered cleanser, and a nonhydrocarbon-based degreasing solution. Residents in intervention apartments were asked not to use Tempo (Bayer HealthCare, LLC, Animal Health Division, Shawnee Mission, KS; a wettable insecticidal powder commonly available, but illegal for consumer sale in New York City), cockroach chalk (Chinese chalk, which is illegal in the United States), or aerosol/spray pesticides. No repeat IPM visits were scheduled, but residents could file complaints per usual practice if they continued to have problems with pests. Only solid or gel baits were applied on those visits in the < 10cases where follow-up occurred.

The control buildings received usual care from the NYCHA’s state-licensed pesticide applicators. Usual care consisted of the offer of conventional, calendar-based extermination visits every 3–6 months, about two-thirds of which resulted in a kitchen baseboard application of spray insecticides containing pyrethroids and the synergist piperonyl butoxide.

### Measures

#### Cockroach and mouse populations

Pest populations were estimated through objective monitoring and sightings reported by residents during the interview. Cockroach populations were monitored by placing five pheromone glue traps in the kitchen for 1 week at baseline (prior to IPM intervention), and 3 months and 6 months later (Chew et al. 2006). It was not always possible to collect all five traps after 7 days, so adjustments were made accordingly. (At least four traps were collected from 95% of apartments at each round of monitoring; traps were left out between 6 and 8 days in 83% of apartments.) Data are reported for the total number of kitchen cockroaches trapped in a week, adjusted for numbers of traps and numbers of days present. We also created dichotomous outcome variables to measure success, defined as having maintained zero pests at follow-up or having reduced the baseline cockroach count by 25% and 50% at 3- and 6-month intervals. We monitored mouse populations using nontoxic, nonrodenticidal bait blocks as previously described (Chew et al. 2006). The presence of mice was analyzed as a dichotomous variable that was considered positive if at least 1 g of bait was consumed from any of the bait blocks placed in the apartment.

During the interview, we asked residents how many cockroaches they had seen, on average, each day over the previous 3 months and assigned responses to one of six categories ranging from 0 to ≥ 20 per day. We also asked how often residents had seen mice, on average, over the same period, with responses assigned to six categories ranging from never to ≥ 5 times each day. We assigned midpoints to the categories to be able to analyze the variable as a count. In addition, we created dichotomous variables to measure pest control success. For subjective roach and mice sightings, success was defined as having maintained zero pests at follow-up or having dropped a specified number of categorical responses. We examined the use of 1, 2, and 3 drops in sighting categories.

#### Allergens

Separate settled dust specimens were collected from the bedrooms (vacuumed from the surface of beds in the upper bed near the pillows) and directly from the kitchen floor at baseline, 3-month, and 6-month intervals as previously described (Chew et al. 2006). Briefly, the sampling equipment included a Mitest dust collector (Indoor Biotechnologies, Charlottesville, VA) attached to a canister vacuum (Eureka Mighty Mite; The Eureka Co., Bloomington, IL) used for 3 min during each sample. Samples were frozen at −20°C for 24 hr immediately after collection. Dust samples were extracted and assayed for mouse urinary protein (MUP) and cockroach [*Blattella germanica* 2 (Bla g 2)] allergens by ELISA. The MUP agents were obtained from Green Laboratories, Inc. (Lenoir, NC) and the Bla g 2 reagents were obtained from Indoor Biotechnologies.

#### Apartment conditions and pesticide use

During the interview, we asked about other factors that could affect pest populations including personal pesticide use, the number of adults and children living in the apartment, open food, clutter, and building maintenance practices.

To assess personal pesticide use, we asked residents whether they had used any of eight different products in the past 3 months, including illegal pesticides (Tempo and Chinese chalk), spray (aerosolized) pesticides, baits, and boric acid. A dichotomous variable for low-toxicity pesticide use was created by evaluating the use of baits, gels, or boric acid. A dichotomous variable for higher-toxicity pesticide use was created by evaluating the use of any illegal or aerosol pesticides. Residents were also asked to rate the quality of building services in their building on a four-point scale from poor to excellent with respect to control of cockroaches and mice.

### Data analysis

Because the IPM intervention was conducted at the building level and because cockroach and mouse presence and allergen levels were quantified in a sample of apartments from each building, we used multilevel modeling to account for the resulting correlation among apartments in the same building. We used SAS statistical software, version 9.1 (SAS Institute Inc., Cary, NC) for all analyses. All references to statistical significance are based on an α of 0.05.

We modeled outcomes in apartments at 3 months and 6 months after baseline. Allergen levels were modeled with linear regression after being log-transformed, using the SAS PROC MIXED procedure. Cockroach counts (unstandardized) were modeled using PROC GLIMMIX, specifying a poisson distribution, and including trap-days (number of traps placed multiplied by days left out) as an apartment-level predictor. The category mid points that were assigned to the subjective cockroach and mouse sightings as count data were also modeled using Poisson regression. We modeled success outcomes and presence of mice with logistic regression, using PROC GLIMMIX, specifying a binomial distribution. Random building intercepts were included in all models, and these intercepts were modeled as a function of IPM status and senior building status. We controlled for baseline levels of pests or allergens and senior building status. We were only able to control for time of the year that apartments were recruited into the study using a dichotomous variable representing the heating season (1 October through 31 May) because the data could not support a finer measure of season.

## Results

Follow-up data were collected from 262 (81%) apartments at 3 months and from 256 (79%) apartments at 6 months. Retention at 3 months was the same in intervention and control groups (81%). Retention at 6 months was slightly lower in intervention apartments compared with control (77% and 82%, respectively). Of the 67 participants lost to follow-up at 6 months, 63% (42) refused to continue participation, 21% (14) had moved, and 16% (11) could not be contacted for other reasons. Overall, 280 apartments provided follow-up data at 3 months or 6 months (87%). We found no significant differences between retained apartments and those lost to follow-up for number of residents or the presence of clutter or open food. Retained apartments were more likely to have had exterminator services within the past 6 months, to have used both safe and unsafe pesticide products, more likely to have had cockroach infestation, less likely to have mice, and more likely to have children < 7 years of age. However, statistical models adjusted for baseline pest or allergen levels and for senior building status.

### Baseline conditions

Table 1 provides baseline descriptions of the 280 apartments for which we had either 3-month or 6-month follow-up data (baseline data for all 323 apartments enrolled in the study have been previously reported) (Chew et al. 2006). Almost one-third of apartments were in senior citizen housing. About half of apartments housed one resident, whereas almost 20% housed four or more. Apartments ranged in size from studios to five-bedroom units. Senior apartments were smaller, typically studios or one-bedrooms. Children < 7 years of age were present in 15% of apartments.

Table 2 describes pests and pesticide use at baseline for the 280 respondents who provided follow-up data. Cockroaches were highly prevalent; 76% of apartments had evidence of cockroaches from traps; 94% were reported by residents to have had cockroaches within 3 months prior to baseline. A sizeable proportion of apartments had extensive infestations: > 100 cockroaches/week were trapped in the kitchens of 35% of apartments, and in 27% of all apartments, residents reported seeing at least 20 cockroaches/day. Because fewer cockroaches were trapped in bedrooms and bathrooms relative to the kitchen, we used kitchen counts as our outcome variable in subsequent analyses of IPM effectiveness. Residents in a large percentage of apartments (89%) reported use of at least one lower-toxicity pesticide product, whereas 64% reported use of at least one higher-toxicity product at baseline.

Objective evidence of mice was detected at baseline in 20% of apartments with follow-up data; in contrast to cockroaches, mice were evident throughout the apartment. Baits were positive for mice in 7% of kitchens, 11% of living rooms, 5% of bathrooms, and 7% of bedrooms. Mouse sightings in the last 3 months were reported by residents in 47% of apartments at baseline, and residents in 16% of apartments reported seeing mice at least daily. There was imperfect correlation between objective and subjective mouse-sighting data. Baits were positive for mice in 8% of apartments where residents reported seeing no mice in the past 3 months and in only 24% of apartments where residents reported seeing mice every day.

### Impact on pests

Table 3 describes the impact of the intervention on cockroach populations. The number of cockroaches trapped in the kitchens of IPM apartments was estimated to be 0.57 times that of control apartments at 3 months [95% confidence interval (CI), 0.33–1.00] and 0.86 times that of control apartments at 6 months (95% CI, 0.56–1.33). Using data based on subjective sightings by residents over the past 3 months, counts of cockroaches in IPM apartment kitchens were 0.44 times those of controls after 3 months of follow-up (95% CI, 0.30–0.64) and 0.55 those of control at 6 months follow-up (95% CI, 0.29–1.05). Adjusting for recruitment time of year as a dichotomous variable had virtually no impact on model results, so it was dropped from analyses.

The odds of successfully reducing counts of trapped cockroaches by 25% and 50% in IPM apartments after 3 months follow-up were 3.1 (95% CI, 1.2–7.8) and 2.7 (95% CI, 1.0–7.8) times the odds in control apartments, respectively (Table 4). The odds of success in IPM apartments after 6 months were also higher than in control apartments. Using decreases in predefined categories of subjective cockroach sightings as measures of success, the odds of a one-category reduction (corresponding to a reduction in daily sightings of 1–10 cockroaches daily, depending on the initial category) in IPM apartments were 3.6 times the odds of success in control apartments (95% CI, 1.8–7.5), whereas the odds of a two-category drop (corresponding to a drop of > 2–20/day) were 3.1 times the odds in control apartments (95% CI, 1.6–5.8).

There was little evidence of success in reducing the presence of mice as measured by bait consumption. Mouse sightings were fewer in IPM compared with control apartments at 3-month and 6-month follow-up, but these differences were not statistically significant (Table 4).

### Impact on allergens

Table 5 shows the ratio of IPM to control group allergen levels per gram of dust. IPM was associated with lower cockroach allergen levels at 6-month follow-up visits in beds and kitchens. For example, the level of Bla g 2 in beds of IPM apartments at the 6-month follow-up visit was 0.4 times the level found in control apartments (95% CI, 0.2–0.8). The IPM apartments also had significantly lower levels of Bla g 2 at the 3-month follow-up visit in the kitchen, but not in the beds. When Bla g 2 was modeled as the absolute amount of allergen per unit area, the IPM group was significantly lower in both the kitchen and bedroom 6 months after the intervention (data not shown). Many of the mouse allergen concentrations for all three time points in beds and kitchens were below the limit of detection (0.5 μg/g), and IPM was not significantly associated with lower levels of mouse allergen at 3 months or 6 months after treatment in either the kitchen or bedroom, whether modeled as MUP per unit area or per gram of dust.

**Figure 1:**
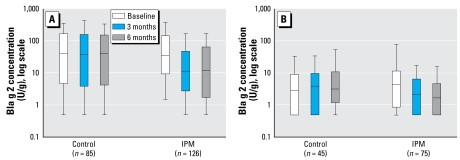
Bla g 2 allergen concentrations (U/g) in kitchens (*A*) and bedroom (*B*) of IPM and comparison apartments at baseline, 3-month, and 6-month time points (box and whiskers represent median, 25–75th, and 10–90th percentiles).

Figure 1 illustrates 10th, 25th, 50th (median), 75th, and 90th percentile levels of Bla g 2 measured in kitchens (Figure 1A) and bedrooms (Figure 1B), on a log scale for the subset of apartments that provided data at all three time points. Kitchen cockroach allergen declined in the IPM group (*n* = 126) at 3 months and were sustained at 6 months, whereas the control apartments (*n* = 85) showed no apparent change. Bedroom median levels of cockroach allergen declined in IPM apartments at both 3 months and 6 months, but rose in control apartments (*n* = 45) over the same time periods.

### Impact on pesticide use

NYCHA pest control staff used no spray pesticides in IPM apartments, in contrast to control apartments. Table 6 summarizes the impact of IPM versus traditional practice on residents’ own use of pesticide products. At baseline, 18% of IPM apartments reported using either Tempo or Chinese chalk to control cockroaches, compared with 8% in control apartments. At 6-month follow-up, only 2% of IPM apartments reported using Tempo or Chinese chalk (a 10-fold drop), whereas use dropped to 5% (half of previous levels) in control apartments at this time point. Use of aerosol pesticides such as sprays, bombs, or foggers dropped in the IPM group from 63% at baseline to 37% after 6 months of follow-up. In control apartments, use of aerosol pesticides barely changed—from 53% at baseline to 59% at 6-month follow-up. Both IPM and control apartments showed substantial reductions in clutter and open food, with little difference between groups. None of these differences were statistically significant.

### Impact on satisfaction with building services

Residents of IPM apartments reported more positive feelings about the quality of NYCHA’s building services at follow-up than those in control apartments (Table 6). In addition, the proportion of residents rating control of cockroaches by the building maintenance staff as poor declined more in IPM apartments than in control apartments, whereas both groups of apartments showed a reduction in the proportion rating mice control as poor. None of these differences were statistically significant.

## Discussion

In this study we sought to determine whether an easily replicable, professionally implemented IPM approach to the eradication and control of residential indoor pests would outperform traditional practices. The search for alternatives to the dependence on higher-toxicity insecticides in residential settings is driven by several factors. The recent epidemic of severe and uncontrolled asthma in urban environments has brought increased knowledge and attention to the problem of indoor allergens from household pests, particularly cockroaches and mice. As awareness grows about the association between indoor pest allergens and asthma symptoms and severity, there is widespread concern in the public health and environmental community that pesticide use will rise. In New York City, > 1,000 accidental exposures to pesticides are reported to the regional poison control center annually, with the vast majority occurring to children in the home (Kass et al. 2005). Although the active ingredients in the baits used in the IPM apartments are mammalian toxicants, by virtue of their solid or gel form, low vapor pressure, placement away from the reach of residents or pets, and the very small quantities used, they pose no significant threat of exposure to residents (National Pesticide Information Center 2009a, 2009b).

The intervention in this study compared the impact of IPM with a traditional pest control approach. The findings show that a single IPM visit was more effective than the regular application of pesticides alone in controlling cockroaches, reducing cockroach allergen levels, reducing resident use of more harmful pesticides by residents, and improving resident satisfaction with building maintenance. IPM was also effective at reducing resident-reported mouse sightings, although the result was not statistically significant, perhaps owing to the small number of buildings in our study and the comparatively low prevalence of mice. These findings are noteworthy for several reasons. First, the IPM intervention reduced both pest populations and allergens relative to traditional pest control. Although kitchen cleaning may have had a direct impact on allergen reductions in kitchens, the fact that allergens were significantly reduced in bedrooms of IPM apartments while those in control apartments were unchanged further demonstrates important benefits from pest interventions in limited areas.

Second, the IPM intervention was delivered with minimal resident education. Most other published studies of IPM that have reported significant impact on pest and allergen levels have involved multiple home visits over 1–2 years to teach residents how to maintain IPM and allergen control (Brenner et al. 2003; Krieger et al. 2005; McConnell et al. 2005; Morgan et al. 2004). Although these approaches appear to be effective at empowering residents to improve their home environment, they are also costly to implement and unlikely to be sustainable by a large public housing authority as a component of routine pest control operations. In contrast, this IPM intervention—completed in a single visit averaging 8–12 person-hours without separate visits to educate residents—may be more replicable. It is difficult to directly compare our impact on pests to other studies because of different monitoring and statistical approaches. In terms of counts of cockroaches, in New York City Brenner et al. (2003) found a 50% reduction at 6 months in cockroach populations in their intervention group and no change in the control group. In contrast, our median weekly kitchen cockroach levels dropped by 75% at 3 months and 88% at 6 months, with increases in control apartments over the same time periods.

Third, the IPM intervention in the present study was successful in decreasing residents’ use of, and potential exposure to, pesticides by reducing the application of spray pesticides by commercial applicators and by reducing the perceived need for the use of aerosol sprays and total release foggers by residents themselves. A noteworthy finding is that traditional pest control had no independent impact over time on objectively determined cockroach levels and minimal impact on resident sightings, suggesting that the use of pesticides alone is both ineffective and an unnecessary introduction of pesticides into the environment, even without an alternative pest control approach to replace it.

There were several limitations of this study. The generalizability of the findings may be limited by the fact that two conditions were considered in the selection of housing developments: *a* ) location in neighborhoods with higher rates of hospitalization for childhood asthma, and *b* ) not having been scheduled for major capital improvements in the following 2 years, suggesting greater structural integrity than some other developments. We did not randomize buildings or apartments to treatment and control status, but rather assigned treatment status to buildings selected to be as similar as possible on potential confounders. Several sources of potential bias exist. We compared the impacts of IPM to quarterly pesticide applications. Normally, a significant proportion of residents refuse or do not make their homes available for these scheduled visits. Given the nature of this study, it is likely the NYCHA reached a greater proportion of control apartments than would otherwise be expected. Also, because buildings were selected for IPM or control status within the same housing development, there was potential for experimental contamination, especially with respect to tenant advice about maintaining pest-free homes and reducing the use of pesticides. Each of these concerns likely biases the results toward the null, potentially leading to an underestimate of true impact. We were unable to fully adjust for time of the year of building recruitment because our adjusted models would only permit inclusion of a dichotomous variable for heating season, which had no impact on results. The impact of recruitment season should be evaluated in future studies of IPM effectiveness.

In conclusion, this study shows that a modest single-visit IPM intervention in public housing is more effective at reducing cockroach populations and allergen levels than traditional scheduled pesticide application alone. In the period of time since the completion of this study, the NYCHA has implemented several key changes to its pest control protocol. The NYCHA has trained its entire workforce of approximately 75 pest control professionals on IPM strategies. It has maintained the IPM unit of nine personnel created for this endeavor, which now carries out identical IPM interventions in severely infested apartments and in apartments during occupant turnover. The NYCHA has suspended the routine use of pyrethroid sprays in all but basement areas not accessible to residents and now applies and distributes cockroach baits during its apartment visits. In addition, the NYCHA has abandoned scheduled calendar visits in favor of visits that account for levels of severity and resident concerns. Where feasible, the NYCHA now orders new kitchen cabinets precaulked and sealed, and its contract specifications for kitchen rehabilitation includes provisions for pest exclusion. The success of the study suggests that this intervention is a model that can be readily replicated by public housing authorities.

## Figures and Tables

**Table 1:** Characteristics of public housing apartments followed for 3 months and 6 months.

	No. of apartments (%)		
Characteristic	All (*n* = 280)	Intervention (*n* = 169)	Control (*n* = 111)
Apartment location			
East Harlem	148 (52.9)	100 (59.2)	48 (43.2)
Brooklyn	132 (47.1)	69 (40.8)	63 (56.8)
Building status			
Family	193 (68.9)	112 (66.3)	81 (73.0)
Senior	87 (31.1)	57 (33.7)	30 (27.0)
No. of residents in the apartment			
1	143 (51.1)	87 (51.5)	56 (50.5)
2	58 (20.7)	35 (20.7)	23 (20.7)
3	25 (8.9)	12 (7.1)	13 (11.7)
4	25 (8.9)	15 (8.9)	10 (9.0)
≥ 5	29 (10.4)	20 (11.8)	9 (8.1)
Apartment is home to children < 7 years of age	42 (15.0)	23 (13.6)	19 (17.1)
No. of floors in the building			
3	62 (22.1)	49 (29.9)	13 (11.7)
6–8	113 (40.4)	63 (37.3)	50 (45.1)
11	48 (17.1)	0 (0.0)	48 (43.2)
20	57 (20.4)	57 (33.7)	0 (0.0)
No. of rooms in apartment			
2	48 (17.1)	32 (18.9)	16 (14.4)
3	95 (33.9)	57 (33.7)	38 (34.2)
4	76 (27.1)	43 (25.4)	33 (29.7)
≥ 5	61 (21.8)	37 (21.9)	24 (21.6)

**Table 2:** Pest sightings, objective pest monitoring results, and pesticide use at baseline.

	No. of apartments (%)		
	All (*n* = 280)	Intervention (*n* = 169)	Control (*n* = 111)
Resident-reported daily cockroach sightings			
Never/<1 per day	55 (19.7)	30 (17.9)	25 (22.5)
1–9/day	109 (39.1)	66 (39.3)	43 (38.7)
10–19/day	40 (14.3)	24 (14.3)	16 (14.4)
≥ 20/day	75 (26.9)	48 (28.6)	27 (24.3)
Cockroaches trapped weekly in the apartment (*n*)			
0	67 (23.9)	38 (22.5)	29 (26.1)
1–10	85 (30.4)	51 (30.2)	34 (30.6)
11–50	62 (22.1)	41 (24.3)	21 (18.9)
51–100	31 (11.1)	15 (8.9)	16 (14.4)
> 100	35 (15.5)	24 (14.2)	11 (9.9)
Mouse sightings in the past 3 months			
Never	146 (52.5)	95 (56.9)	51 (45.9)
Less than once/week	58 (20.9)	29 (17.4)	29 (26.1)
1–6 times/week	29 (10.4)	16 (9.6)	13 (11.7)
At least once/day	45 (16.2)	27 (16.2)	18 (16.2)
Baits positive for mice in the apartment	36 (19.7)	22 (20.7)	14 (18.2)
Use lower-toxicity pesticides			
Containerized baits	211 (75.4)	128 (75.7)	83 (74.8)
Boric acid	93 (33.2)	58 (34.3)	35 (31.5)
Gel baits	123 (43.9)	69 (40.8)	54 (48.7)
Sticky traps	98 (35.0)	69 (40.8)	29 (26.1)
Use higher-toxicity pesticides			
Bombs/foggers	25 (8.9)	15 (8.9)	10 (9.0)
Chalk	17 (6.1)	14 (8.3)	3 (2.7)
Sprays	162 (57.9)	104 (61.5)	58 (52.3)
Tempo	31 (11.1)	24 (14.2)	7 (6.3)

**Table 3:** Effectiveness of IPM intervention after 3 months and 6 months follow-up on cockroaches (trapped and sighted).

		3-month follow-up		6-month follow-up	
Weekly cockroach counts	Baseline median (range)	Median (range)	Adjusted^a^ IPM: control count ratio (95% CI)	Median (range)	Adjusted^a^ IPM: control count ratio (95% CI)
Trapped in kitchen^b^					

IPM	8 (0–373)	2 (0–212)	0.57 (0.33–1.00)	1 (0–161)	0.86 (0.56–1.33)
Control	3 (0–450)	7 (0–357)	1.00 (reference)	5 (0–330)	1.00 (reference)

Sighted in apartment					
IPM	53 (0 to > 140)	25 (0 to > 140)	0.44 (0.30–0.64)	7 (0 to > 140)	0.55 (0.29–1.05)
Control	53 (0 to > 140)	53 (0 to > 140)	1.00 (reference)	25 (0 to > 140)	1.00 (reference)

a Adjusted for baseline cockroach counts and senior building status; trapped cockroach counts also adjusted for number of trap-days.

b Trapped cockroach counts are standardized to five traps placed for 7 days; sighted cockroach counts are assigned the midpoint of numeric category values.

**Table 4:** Success of IPM in reducing or maintaining zero cockroach and mice populations.

	Success at 3 months^a^			Success at 6 months^a^		
	IPM apartments No. (%)	Control apartments No. (%)	Odds ratio IPM: control (95% CI)	IPM apartments No. (%)	Control apartments No. (%)	Odds ratio IPM: control (95% CI)
Count of cockroaches trapped						
Model 1: 25% reduction or remaining zero	121 (77)	56 (54)	3.1 (1.2–7.8)	113 (76)	62 (58)	2.1 (1.0–4.5)
Model 2: 50% reduction or remaining zero	108 (68)	50 (48)	2.7 (1.0–7.8)	101 (68)	57 (54)	1.7 (0.7–3.9)

Sighted cockroaches						
Model 3: drop of > 1 category or remaining zero	97 (62)	32 (31)	3.6 (1.8–7.5)	97 (66)	44 (42)	2.5 (0.7–9.2)
Model 4: drop of > 2 categories or remaining zero	57 (36)	16 (15)	3.1 (1.6–5.8)	65 (44)	18 (17)	3.0 (0.7–1.2)

Mice activity						
Model 5: no mouse activity	141 (90)	90 (88)	1.2 (0.4–3.5)	130 (88)	92 (87)	1.0 (0.2–4.9)

Sighted mice						
Model 6: drop of > 3 categories or remaining zero	119 (76)	61 (59)	3.6 (0.9–14.7)	109 (74)	55 (52)	3.7 (0.6–22.1)

a Cockroach and mouse success models are adjusted for baseline cockroach counts and senior building status, except for Model 3, which could not be fit with senior building status.

**Table 5:** Ratio of allergen levels in IPM to control apartments at 3-month and 6-month follow-up.^a^

Allergen/Outcome	IPM:control ratio	95% CI
Bla g 2 (U/g)		
Bedroom: 3-month follow-up	0.6	0.3–1.3
Bedroom: 6-month follow-up	0.4	0.2–0.8
Kitchen: 3-month follow-up	0.4	0.2–0.8
Kitchen: 6-month follow-up	0.3	0.1–0.9

MUP (μg/g)		
Bedroom: 3-month follow-up	0.9	0.6–1.3
Bedroom: 6-month follow-up	0.8	0.6–1.1
Kitchen: 3-month follow-up	0.7	0.4–1.5
Kitchen: 6-month follow-up	0.7	0.4–1.4

a Multiple regression model for predicting allergen concentration at follow-up evaluations adjusted for baseline allergen levels and senior building status.

**Table 6:** Impact of IPM after 3 months and 6 months on residents’ personal use of pesticides and perceptions of building maintenance.

	Baseline (%)	3 months (%)	6 months (%)
Households reporting			
Use of Tempo or chalk			
IPM	18.1	1.9	2.0
Control	8.1	8.7	4.8
Use of aerosol pesticides			
IPM	62.7	38.6	36.5
Control	53.2	53.9	49.5

Household rating			
Cockroach control as poor			
IPM	52.5	21.8	26.4
Control	44.4	40.6	44.2
Mouse control as poor			
IPM	36.7	26.5	21.6
Control	37.1	31.0	36.2
